# Traumatic Finger Amputations

**Published:** 1912-06

**Authors:** Alfred H. Noehren

**Affiliations:** Buffalo, N. Y.


### Traumatic Finger Amputations.

ALFRED H. NOEHREN, M. D.
Buffalo, N. Y.

THIS paper is based on a series of 55 traumatic finger amputations in 35 different patients which came under the care of the writer from January 1, 1909 to March 1, 1912. All were the result of industrial accidents, the majority of the pati-

* Read before the Buffalo Medical and Surgical League, May 9, 1912.

ents having had their fingers caught in stamping machines powerful enough to go through a steel plate, cutting off the fingers with little injury to the remainder of the hand. The rest were due to crushing injuries, tearing the skin in all directions, grinding up the tendons and muscles and fracturing or crushing the bones. Most of the men had their hands covered with oil, soot, and dust, or wore dirty gloves at the time of the injury and these were ground into the wound.

The cases in which one or two fingers only were involved were treated under local anesthesia in the office. The remainder were taken to the Deaconess Hospital and a general anesthetic administered. In one case three fingers were amputated in the office, but it was decided at the time that the pain and ordeal were greater than could reasonably be asked and since then two fingers have been the limit for local anesthesia in the office, other things being equal.

In three cases, secondary amputations had to be done, because treated too conservatively at first. In the one, the middle finger was crushed and an attempt made to save the finger, but gangrene developed and necessitated amputation at the metacarpo-phalangeal joint. In the second case it was attempted to save a crushed distal phalanx, but nine days later it had to be amputated on account of sloughing and gangrene. In the third case, enough of the phalanx had not been removed to allow the soft parts to cover the bone. About one-quarter inch was later removed and the finger promptly healed up. In a fourth case the patient would not consent to the removal of any bone, because it did not project beyond the soft parts, but returned in three weeks and asked that it be done. By this time the soft parts had retracted, leaving a tender stump of bone projecting.

In treating these cases in the office, it has been the writer's aim to develop a method and technique that would as far as possible satisfy the following conditions: 1. simplicity, 2. minimum of pain and shock to patient, 3. preservation of as much useful stump as possible, 4. minimum time for healing and disability of patient.

After trying various methods, the following has given the greatest satisfaction.

### PREPARATION OF PATIENT.

1. Tying of rubber catheter around base of finger, or, if necessary, an Esmark bandage around the wrist, to control hemorrhage and increase the action of the local anesthetic.

2. Cleansing of skin surrounding wound with turpentine.

3. Cleansing of wound by pouring hydrogen peroxide over it rather liberally a few times.

4. Drying of wound and surrounding skin with ether.

5. Painting of wound and skin with Tincture of Iodine. While the iodine is acting, the instruments may be boiled and the hands of the operator scrubbed up and immersed a few minutes in bichloride of mercury.

6. Protection of wound by cutting slit in piece of sterile gauze, slipping it over the stump, and covering rest of hand with sterile gauze.

OPERATION.

1. Injection of syringeful of Quinine and Urea Hydrochloride into the wound, especially into that part of the skin where an incision is to be made and close to and around the bone, where the chief dissection takes place. Some time must be given for this to act.

2. Dissection of as much bone as is necessary to allow covering of the remainder by the soft parts. Unless the skin is already torn, this is best accomplished by making a lateral incision on one or both sides directly down to the bone, thus making an anterior and posterior flap. In dissecting, the bone is hugged closely and either the scalpel or scissors used. In this way, infection and destruction of the soft parts are reduced to a minimum. After freeing enough bone, usually about one-quarter inch from the extremity of the soft parts, the periosteum is scraped back and all the tissues held out of the way with a thumb forceps.

3. Removal of bone with the bone-forceps and trimming of edges with bone-forceps and scissors. This seldom causes the least pain.

If the injury is just distal to a joint, it is better to amputate at the joint, except in the case of the proximal phalanx, where even the shortest stump is of great value. In amputating at an interphalangeal joint, dissection is done along the dorsum of the bone to the joint, the joint is forcibly flexed, the capsule punctured with the scalpel, and the anterior surface of the bone dissected from the joint outward.

4. Covering of bone stump with two to four through-and-through silkworm-gut sutures, including the flexor and extensor tendons, subcutaneous tissue, and skin. The periosteum or joint capsule may also be included, if possible, but whether or not this was done seemed to make little difference in the results.

5. Insertion of small gauze drain down to the bone. If there is sufficient gaping between the stitches, this is not necessary.

6. Covernig of wound with a tight temporary gauze dressing to control hemorrhage and removal of tourniquet. After about five minutes, this dressing is removed, hydrogen peroxide once more poured over the wound, and a dry gauze dressing rather snugly applied. A slight oozing may be disregarded. The patient should be instructed not to remove the dressing, but to cover it with a clean cloth, if blood comes through.

It will be noted that in this operation no catgut is used and the tissues are not sutured in layers, periosteum, tendons, and skin, but by through-and-through silkworm-gut sutures. These are boiled with the instruments, are easily handled, and later easily removed. This prevents infection and makes the operation much shorter and simpler. Formerly the writer carefully sewed the periosteum or joint-capsule and the tendons with catgut, but found the results better with the simpler and easier way. The same is true of tying off blood-vessels. A great deal of time may be used and much damage to tissues done in looking for blood-vessels and in tying them more catgut is buried in the wound, thus increasing still more the chance of infection, while the method described above controlled the hemorrhage in every case done in the office.

All, this, however, does not hold good in the more extensive injuries to the hand operated upon under general anesthesia in a hospital with plenty of assistance or even in amputation of a finger for other reasons than for injury to a dirty hand in a dusty shop.

### AFTER-TREATMENT.

The patients are instructed to return in two days. If there should be much pain, they are to return the following day. The dressing is then loosened by immersing it in dilute hydrogen peroxide and removed. The drain, if present, is also removed. If the wound looks good, a small amount of antiseptic powder is dusted on the wound and another gauze dressing applied. If the wound looks inflamed, a moist 1-5,000 bichloride dressing is put on and the patient instructed to keep it moist. The stitches are removed on the fourth or fifth day.

### RESULTS.

Complete healing in these office cases usually took place in ten days. Some were healed in one week and some required two weeks. Several patients returned to work the next day and most of them in the course of a week. No case of painful scar or nerve-ending was seen, although the patients were asked to return, if any trouble developed. The motion of the stump was good in every case, as was also the cosmetic result.

Lest it be inferred that not sufficient conservatism was practiced in these cases, the writer wishes to state that during this same period, five cases of compound fracture of a phalanx were treated conservatively with splint and sewing of lacerated soft parts with good results. On the other hand, to be too anxious to save a finger that in the end may be stiff and useless or may have to be amputated anyway with a long period of disability, is not always doing a working-man the most good.

The more extensive cases operated at the hospital will not be described, as it is the purpose of this paper rather to present a few practical points learned from the treatment of the kind of finger injury that is liable to present itself in the office of every practicing physician.

519 E. UTICA STREET.

